# Infective Endocarditis Associated With Intravenous Drug Use: Clinical Course, Biological Characteristics, and Outcomes of a Romanian Cohort

**DOI:** 10.7759/cureus.96215

**Published:** 2025-11-06

**Authors:** Adina A Nanu, Miruna I Lazar, Simin A Florescu

**Affiliations:** 1 Infectious Diseases, Carol Davila University of Medicine and Pharmacy, Bucharest, ROU; 2 Infectious Diseases, Victor Babeș Clinical Hospital for Infectious and Tropical Diseases, Bucharest, ROU; 3 Allergy and Immunology, Nicolae Malaxa Clinical Hospital, Bucharest, ROU

**Keywords:** infective endocarditis, in-hospital mortality, injection drug use, people who inject drugs, staphylococcus aureus

## Abstract

Background

Injection drug use is causing a wave of infective endocarditis (IE) worldwide, a severe and potentially deadly infection of the heart’s endothelium. While this important public health problem has been well described in Western countries, available data from Eastern Europe are quite limited. Our study set out to describe this particular patient profile and its background, the clinical course of IE in this population, and evolution during and after hospitalization in a major infectious disease hospital in Bucharest, Romania.

Methodology

We reviewed the patient records of 57 people who inject drugs (PWID) admitted with IE between August 2019 and July 2025. Clinical, microbiological, and outcome data were collected and analyzed using standard descriptive statistics.

Results

The patients were mostly young men with a median age of 34 years. Most of these patients used heroin and other psychoactive substances and faced severe chronic co-infections: 63% were human immunodeficiency virus-positive, and 98% had hepatitis C. Most patients presented to the hospital with fever, malaise, and respiratory symptoms. Echocardiography confirmed valvular vegetations in over 90% of cases, most frequently on the tricuspid valve. Blood cultures were positive in 70%, most often revealing *Staphylococcus aureus* bacteriemia. More than half had embolic complications, 30% needed intensive care, and a quarter were candidates for cardiovascular surgery. The median hospital stay was 28 days, while mortality remained high at 17.5% during hospitalization and 35% at one year.

Conclusions

IE among PWID represents a life-threatening illness, particularly affecting young adults with multiple infections and comorbidities who are additionally burdened with social challenges. Despite prolonged hospitalizations and intensive care, their short- and long-term outcomes remain unfavorable.

## Introduction

The global increase in injection drug use has led to a substantial burden of opioid and methamphetamine use disorders, overdose deaths, and infectious complications, including drug use-associated infective endocarditis (DUA-IE) [1]. Hospitalizations for DUA-IE are rising, sometimes steeply, in young adults, and managing these infections is especially difficult given their high morbidity and mortality, as well as ease of reinfection [1]. Therefore, successful management requires not only antimicrobial and surgical interventions but also care of the underlying substance use disorder and psychosocial complexities, which are ideally delivered by multidisciplinary teams, including addiction medicine, cardiology, infectious diseases, cardiac surgery, and psychiatry [2]. Despite these approaches, DUA-IE now accounts for nearly one-third of all valve surgeries for infective endocarditis (IE) in the United States, with a high risk of recurrence and difficult decisions regarding reoperation [3].

IE remains a major cause of morbidity and mortality, with a stable overall incidence in recent decades. However, a clear rise has been observed among people who inject drugs (PWID) [4]. In this particular population, IE typically affects younger individuals, is strongly associated with *Staphylococcus aureus*, and often involves right-sided heart valves and pulmonary septic emboli [5]. PWID now account for an increasing proportion of community-onset IE, representing up to 16% of cases in North America, with hospitalization rates and healthcare costs rising sharply; recurrent IE is common, and injection drug use itself is the strongest predictor of reinfection [6]. Compared with non-PWID, these patients face a 50-100-fold higher risk of developing IE, and despite guideline-based indications for surgery, long-term outcomes remain poor, with high reinfection and reoperation rates that raise ongoing ethical debates regarding repeat surgical interventions [7].

PWID are at high risk for IE, a serious infection of the heart valves that can be deadly and often requires long hospital stays or surgery. In the United States, cases are rising, especially among young, white women in rural areas [8]. PWID with IE are usually younger and healthier than non-PWID but face more recurrences and often right-sided valve involvement, with *Staphylococcus aureus* as a common cause [8,9]. Surgery can help, yet doctors sometimes hesitate due to the risk of reinfection [8]. In Europe, PWID account for around 10% of IE cases, showing this represents a major problem [9].

The opioid crisis has caused devastating mortality in the United States, with around 130 deaths per day from prescription overdoses. Opioid use and misuse are also rising in Europe, though at lower levels. As opioid prescribing and related hospitalizations increase, especially in countries such as the Netherlands, this trend is expected to contribute further to injection drug use and its infectious complications, including IE [10].

Most of the research and monitoring in PWID has focused on chronic infections such as HIV, hepatitis B, and hepatitis C. In Romania, recent data show that around 16-20% of PWID are human immunodeficiency virus (HIV)-positive, about 8-10% have hepatitis B virus (HBV), and more than 60% live with hepatitis C virus (HCV). These numbers underline the heavy burden of viral infections in this group [11]. By comparison, bacterial complications such as IE are much less studied, even though cases are increasing and outcomes are often severe.

## Materials and methods

Study design

This descriptive, prospective study was conducted at the Clinical Hospital of Infectious and Tropical Diseases “Dr. Victor Babeș” in Bucharest between August 2019 and July 2025. It aimed to highlight the clinical, microbiological, and outcome characteristics of 57 adult patients belonging to the category of PWID, who were hospitalized with IE during this period. These patients were selected through a systematic review of the hospital’s electronic database using international diagnostic codes (ICD-10) corresponding to IE, such as I33, I38, I39, along with personal behavioral history of active or prior intravenous (IV) drug use in each patient’s medical chart. Each medical record was thoroughly reviewed manually to confirm diagnosis according to the modified Duke criteria. Diagnosis was established according to the Duke criteria valid at the time of hospitalization. Patients admitted before 2023 were evaluated using the original Duke criteria, while those admitted in 2023 or later were assessed using the updated Duke-International Society for Cardiovascular Infectious Diseases (Duke-ISCVID) criteria.

We collected clinical, laboratory, microbiological, echocardiographic, and outcome information from both electronic and paper records using a Microsoft Excel 2018 database. The variables included basic demographics, IV drug use history, comorbidities, HIV/hepatitis B and C status, clinical presentation, microbiology results, echocardiography findings, laboratory values at admission and discharge, in-hospital treatment, complications, and outcomes. When specific information was missing, it was marked as such and not available (NA).

Objectives

This study aims to describe both the clinical and paraclinical (including microbiological) characteristics and evolution of IE among PWID treated in our hospital in Bucharest. We acknowledged the need for a better picture of this specific patient profile, as well as their behavioral background, clinical presentation, and microbiological etiology. We were also interested in their in-hospital management and outcomes, specifically complications and mortality (short-term and one-year mortality). In other words, we wanted to put together a clearer picture of IE in PWID in our setting in Eastern Europe and to contribute to a better understanding of this public health issue.

Admission and diagnostic procedures

At admission, all of the patients were assessed by the on-call team for clinical and paraclinical signs of systemic infection. Clinical suspicion of IE was based upon both medical and behavioral history, symptoms and their onset, laboratory abnormalities, and rapidly available imaging studies when indicated (chest X-ray).

In accordance with internal hospital protocols and regulations, three sets of blood cultures, each consisting of one aerobic and one anaerobic sample, were collected at least one hour apart, preferably before the initiation of empirical IV antibiotic therapy.

Diagnoses were established according to the Duke criteria valid at the time of hospitalization: patients who were admitted before 2023 were evaluated using the original Duke criteria, while those hospitalized in 2023 or later were assessed using the updated Duke-ISCVID criteria. To ensure diagnostic consistency across the entire study period, all of the included cases were retrospectively reclassified using the updated Duke-ISCVID criteria, using online tools such as the MEDcalc calculator. Cases were categorized as definite, possible, or rejected IE, but only definite and possible cases of IE were finally included in the statistical analysis, as both groups benefited from identical clinical management.

Comorbidities were evaluated using the Charlson Comorbidity Index, based on documented diagnoses at admission, supported by laboratory or imaging results when available, following the original method described by Charlson et al. The Sequential Organ Failure Assessment (SOFA) score was calculated using the first clinical and laboratory values recorded within the first 24 hours of admission.

Blood cultures were processed according to the standard microbiology procedures used in our hospital during the study period. Microorganisms were identified using automated systems (such as VITEK 2), and antibiotic susceptibility was interpreted according to the European Committee on Antimicrobial Susceptibility Testing guidelines that were current at the time of testing. For patients with consistently negative blood cultures, an extended diagnostic workup for culture-negative IE was conducted, and, when clinically appropriate, serological tests for *Coxiella burnetii* and *Bartonella spp*. were obtained in line with institutional protocol, an approach that had already been implemented before the updated Duke criteria were introduced. Echocardiography (transthoracic or transesophageal) was performed by certified cardiologists using uniform institutional protocols, and reports were reviewed to document valve involvement, vegetation size, and possible complications such as abscesses or regurgitation.

Patients were followed for one year after discharge by checking outpatient records, rehospitalization data, and, when possible, direct contact. Recurrence was defined as a new episode meeting Duke criteria after completing antibiotic treatment and at least six months after discharge. Patients who left the hospital against medical advice were still included in the outcome analysis when follow-up information was available.

Inclusion criteria

**Figure 1:**
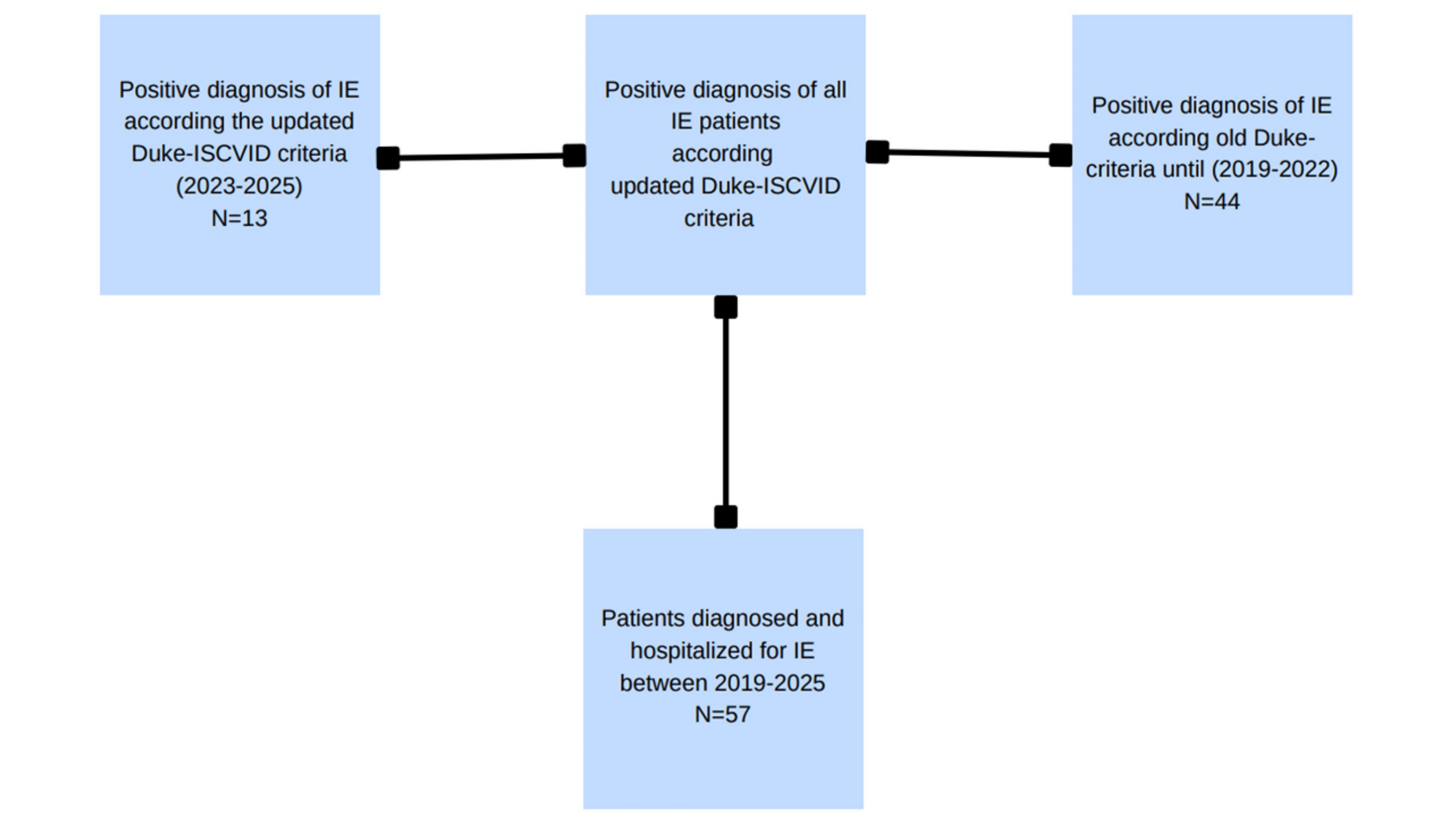
Flowchart showing the criteria of inclusion. Duke-ISCVID= Duke-International Society for Cardiovascular Infectious Diseases; IE = infective endocarditis

Inclusion criteria comprised PWID diagnosed with definite or possible IE according to the modified Duke criteria from August 2019 to July 2025 who offered their written informed consent regarding data collection for research purposes. Figure 1 depicts a flowchart of the patient inclusion process.

Exclusion criteria

We excluded patients under 18 years old, who did not offer their informed consent, and who were diagnosed with IE, but were not PWID.

Statistical analysis

Our patient data were collected and organized in a Microsoft Excel 2018 database. Categorical variables were described as absolute (n) and relative frequencies (%). Continuous variables were summarized using means ± standard deviations (SDs) and medians (interquartile ranges, IQRs). We also performed paired comparisons for non-parametric data (laboratory data changes from admission to discharge) using the Wilcoxon signed-rank test using SPSS Statistics version 26 (IBM Corp., Armonk, NY, USA). All p-values under 0.05 were considered statistically significant. Variables with missing values were continuous and were analyzed based on the available data only, according to their distribution. Subgroup or regression analyses were not conducted due to sample size limitations.

## Results

Demographic characteristics

**Figure 2:**
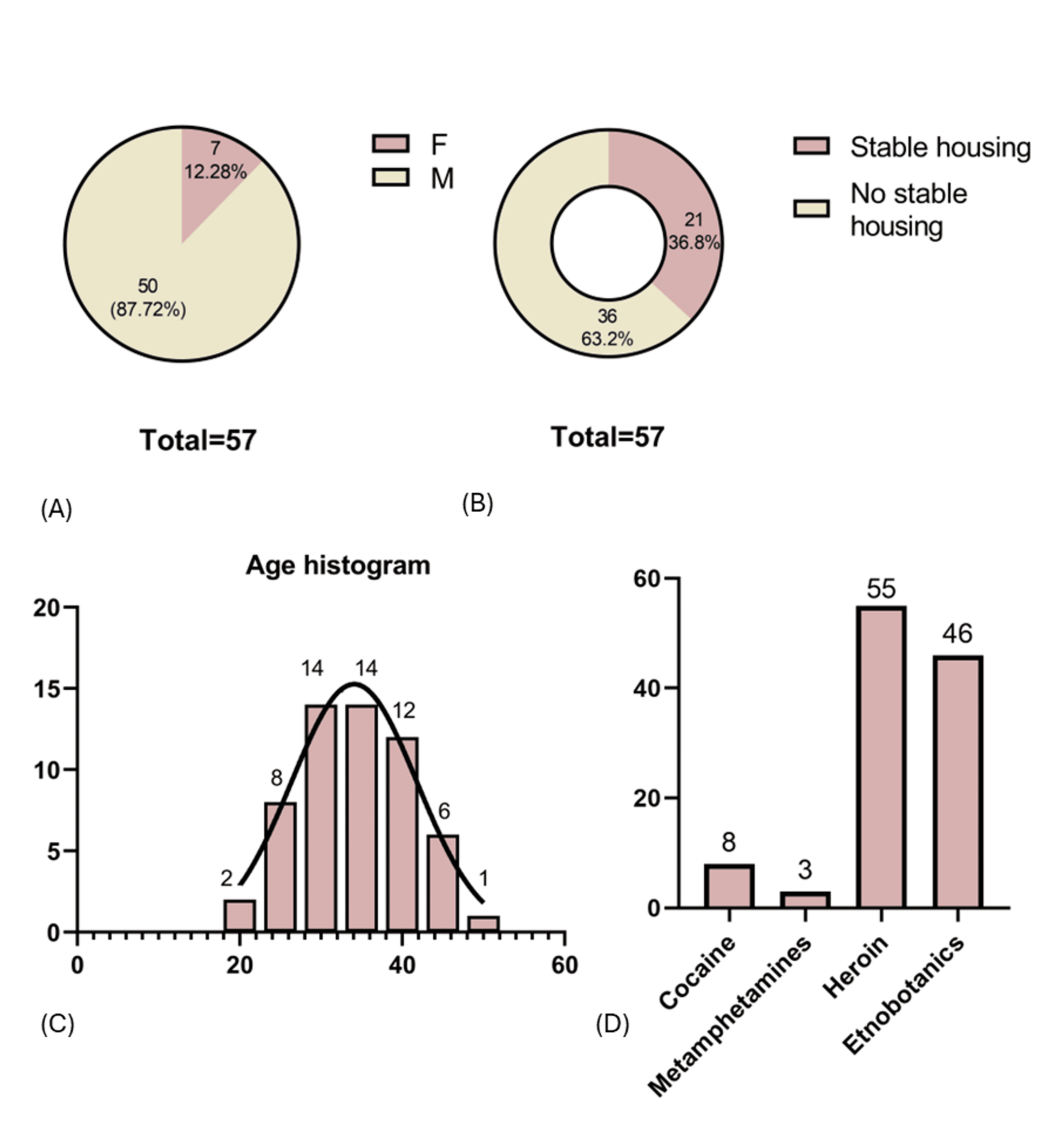
Demographic distribution, housing stability, and drug use profile among PWID (N = 57). (A) Sex distribution, showing a male sex predominance. (B) Proportion of participants without stable housing. (C) Age distribution histogram; the Gaussian curve shows normal distribution and a predominance of 30-35-year-old patients. (D) Frequency of reported use of major substances, showing a high prevalence of heroin and ethnobotanic use. PWID = people who inject drugs

Nearly 88% of patients were of the male sex, with a median age of 34 years. About two-thirds reported stable housing. Drug use had started early, around 21 years of age, and continued for more than 12 years on average. Almost all patients used heroin (96%) and new psychoactive substances (81%), while cocaine and methamphetamine were less frequent. Three-quarters had injected drugs within one week before admission. Nearly one-third were on methadone substitution therapy. Tobacco use was universal, and 88% also reported alcohol use, mostly occasional. These findings are presented below in Table 1 and Figure 2.

**Table 1:** Demographic profile, housing stability, and substance use patterns among PWID (N = 57). SD = standard deviation; IQR = interquartile range; IV = intravenous; PWID = people who inject drugs

Parameter	PWID (N = 57)
Sex	
Females, n (%)	7 (12.3%)
Males, n (%)	50 (87.8%)
Age (years)	
Mean ± SD	34.32 ± 6.69
Median (IQR)	34 (29–40)
Stable housing, n (%)	36 (63.2%)
Cocaine usage, n (%)	8 (14%)
Methamphetamine usage, n (%)	3 (5.3%)
Heroin usage, n (%)	55 (96.5%)
Etnobotanics usage, n (%)	46 (96.5%)
Duration of IV drug usage (years)	
Mean ± SD	12.55 ± 4.99
Median (IQR)	12.5 (8.25–15)
Age at first IV drug use (years)	
Mean ± SD	21.46 ± 6.33
Median (IQR)	21 (17–25.75)
IV drug administration one week prior, n (%)	43 (75.4%)
Actual methadone substitution, n (%)	17 (29.8%)
History of methadone substitution, n (%)	15 (26.3%)
Smoking, n (%)	57 (100%)
Alcohol consumption, n (%)	50 (87.7%)
Occasional alcohol consumption, n (%)	34 (68%)
Daily alcohol consumption, n (%)	16 (32%)

Personal history and comorbidities

As expected for this particular population, infectious comorbidities were very common in our study group. Overall, 63% of patients were HIV-positive, many with advanced disease stages. Almost all had chronic HCV infection (98%), while HBV (7%) and syphilis (5%) were less frequent. Non-infectious comorbidities were recorded in about one-quarter of cases, as shown in Table 2.

**Table 2:** Infectious disease history, comorbidity burden, and clinical severity assessment among PWID (N = 57). SD = standard deviation; IQR = interquartile range; IV = intravenous; PWID = people who inject drugs; SOFA = Sequential Organ Failure Assessment; HIV = human immunodeficiency virus; HBV = hepatitis B virus; HCV = hepatitis C virus; IE = infective endocarditis

Parameter	PWID (N = 57)
Charlson Comorbidity Index (points)	
Mean ± SD	5.35 ± 3.18
Median (IQR)	7 (1–7)
SOFA score (points)	
Mean ± SD	2.33 ± 2.25
Median (IQR)	2 (0–3)
History of HIV infection, n (%)	36 (63.2%)
B1 stage	3 (8.33%)
B2 stage	9 (25%)
B3 stage	11 (30.5%)
C2 stage	1 (2.7%)
C3 stage	12 (33.3%)
History of HBV, n (%)	4 (7%)
History of HCV, n (%)	56 (98.2%)
History of Treponema pallidum infection (syphilis), n (%)	3 (5.3%)
History of IE (>6 months prior), n (%)	8 (14%)
Years since last IE episode	
Mean ± SD	4.37 ± 4.41
Median (IQR)	2 (1–8)
Non-infectious comorbidities, n (%)	13 (22.8%)

Clinical presentation and imaging data

**Figure 3:**
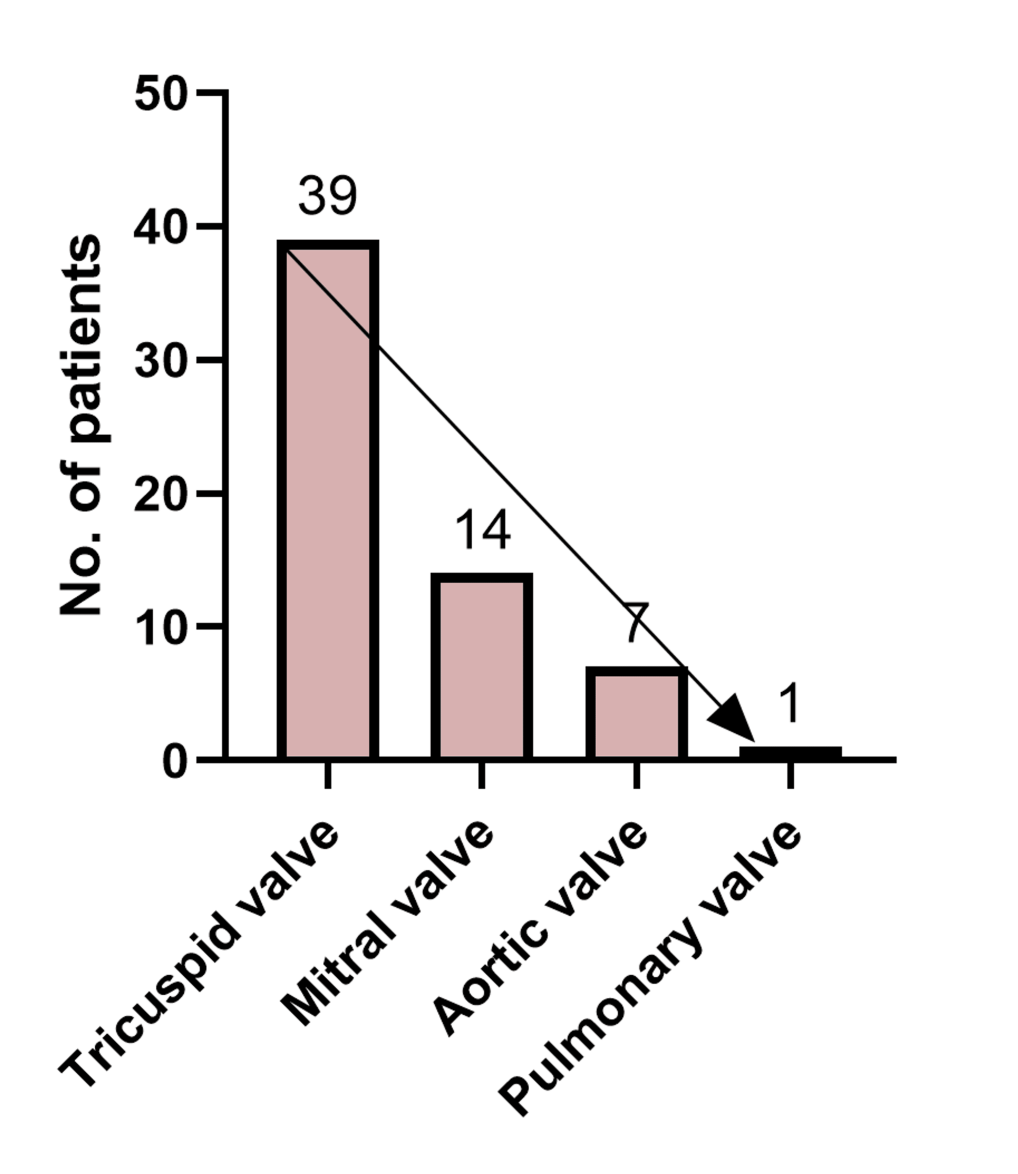
Bar chart illustrating the prevalence of affected valves.

Most of the patients presented with fever (97%), fatigue (97%), and weakness (91%). Cough (75%) and dyspnea (42%) as respiratory symptoms were also common. On physical examination, murmurs were heard in nearly two-thirds, and hepatomegaly was noted in 74%. Chest X-ray showed pulmonary infiltrates in 72% and pleural effusion in 30% (Table 3). Echocardiography detected vegetations in more than 90% of cases, most often on the tricuspid valve (68%), followed by mitral (25%) and aortic (12%) valves (Figure 3). Only a few had pre-existing valvular disease (Table 4).

**Table 3:** Clinical signs, symptoms, and vital parameters at admission among PWID (N = 57). SD = standard deviation; IQR = interquartile range; PWID = people who inject drugs

Parameter	PWID (N = 57)
Fever, n (%)	55 (96.5%)
Chills, n (%)	34 (59.6%)
Maximal temperature during hospitalization (°C)	
Mean ± SD	38.82 ± 0.7
Median (IQR)	38.8 (38.2–39.4)
Profuse sweating, n (%)	20 (35.1%)
Dyspnea, n (%)	24 (42.1%)
Palpitations, n (%)	5 (8.8%)
Cough, n (%)	43 (75.4%)
Edemata, n (%)	13 (22.8%)
Peripheral, n (%)	9 (15.8%)
Precordial pain, n (%)	5 (8.8%)
Malaise, n (%)	52 (91.2%)
Myalgias, n (%)	16 (28.1%)
Arthalgias, n (%)	7 (12.3%)
Fatigability, n (%)	55 (96.5%)
Lack of appetite, n (%)	51 (89.5%)
Chest pain, n (%)	9 (15.8%)
Syncope, n (%)	7 (12.3%)
Systolic arterial pressure ≤90 mmHg, n (%)	9 (15.8%)
Heart rate (beats/minute)	
Mean ± SD	101.47 ± 17.7
Median (IQR)	100 (90–115)
Respiratory rate (breaths/minute)	
Mean ± SD	23.32 ± 7.07
Median (IQR)	21 (18–28.5)
SpO_2_ (%)	
Mean ± SD	94.81 ± 4.73
Median (IQR)	97 (94–98)
Rales, n (%)	27 (47.4%)
Crepitants, n (%)	9 (33.3%)
Bronchial, n (%)	18 (66.6%)
Systolic murmur, n (%)	37 (64.9%)
Diastolic murmur, n (%)	3 (5.3%)
Swollen lymph nodes, n (%)	35 (61.4%)
Abdomen tenderness, n (%)	21 (36.8%)
Diffuse, n (%)	16 (76.2%)
Epigastric region, n (%)	4 (7.01%)
Right upper quadrant, n (%)	1 (4.8%)
Hepatomegaly, n (%)	42 (73.7%)
Diarrhea, n (%)	7 (12.3%)
Focal neurological signs, n (%)	2 (3.6%)
Meningitis signs, n (%)	5 (8.8%)
Alveolar lung disease on X-ray, n (%)	41 (71.9%)
Pleural fluid on X-ray, n (%)	17 (29.8%)

**Table 4:** Echocardiographic findings, valve involvement, and IE diagnostic criteria among PWID (N = 57). IE = infective endocarditis; PWID = people who inject drugs; TTE = transthoracic echography; TEE = transesophageal echography; TV = tricuspid valve; MV = mitral valve; AV = aortic valve; PV = pulmonary valve; IAS = interatrial septum; CHF = congestive heart failure

Parameter	PWID (N = 57)
Bioprosthetic valve IE, n (%)	1 (1.8%)
Native valve IE, n (%)	55 (96.5%)
Interatrial septum IE, n (%)	1 (1.8%)
Preexistent valvulopathy, n (%)	3 (5.3%)
Mitral, n (%)	1 (33.3%)
Tricuspid, n (%)	2 (66.6%)
Aortic, n (%)	0 (0%)
Pulmonary, n (%)	0 (0%)
Preexistent valvular insufficiency, n (%)	3 (5.3%)
Preexistent valvular stenosis, n (%)	0 (0%)
Identified valvular vegetations, n (%)	52 (91.2%)
TTE, n (%)	52 (91.2%)
TEE, n (%)	3 (5.3%)
IE on TV, n (%)	39 (68.4%)
IE on MV, n (%)	14 (24.6%)
IE on AV, n (%)	7 (12.3%)
IE on PV, n (%)	1 (1.8%)
EI IAS, n (%)	1 (1.8%)
Vegetation size, longitudinal (cm), median (IQR)	1.5 (1.2–1.875)
Vegetation size, transverse (cm), median (IQR)	1 (0.5–1.475)
Secondary vegetation size, longitudinal (cm), median (IQR)	1.25 (0.7–2.62)
Secondary vegetation size, transverse (cm), median (IQR)	0.8 (0.425–1.85)
More than one valve affected, n (%)	4 (7%)
Certain IE according to renewed Duke criteria, n (%)	50 (87.7%)
1 Duke major criterion, n (%)	20 (35.1%)
2 Duke major criteria, n (%)	36 (63.2%)
CHF, n (%)	8 (14%)

Microbiological findings

**Figure 4:**
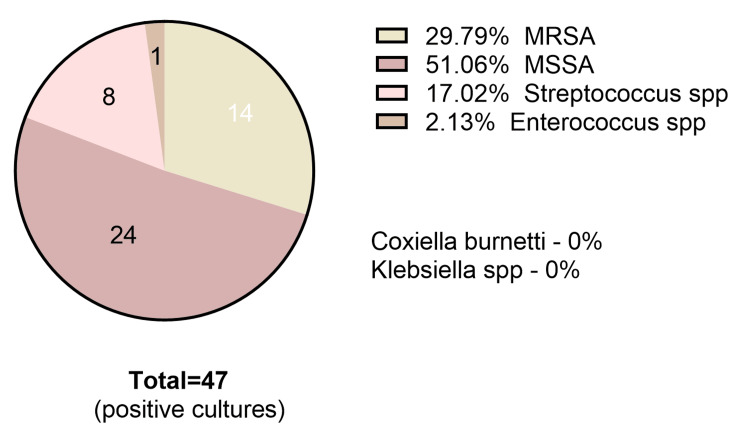
Pie chart describing the prevalence of IE with each identified pathogen. IE = infective endocarditis; MRSA = methicillin-resistant *Staphylococcus aureus*; MSSA = methicillin-sensitive *Staphylococcus aureus*

Blood cultures were positive in 70%. *Staphylococcus* species were the most frequent pathogens (67%), with both methicillin-resistant *Staphylococcus aureus* (37%) and methicillin-sensitive *Staphylococcus aureus* (63%). Streptococci were isolated in 14%. Fungal infections occurred in 11%, and 4% had polymicrobial infections (Figure 4, Table 5).

**Table 5:** Microbiological profile and identified pathogens among PWID with IE (N = 57). IE = infective endocarditis; PWID = people who inject drugs; MRSA = methicillin-resistant *Staphylococcus aureus*; MSSA = methicillin-sensitive *Staphylococcus aureus*

Parameter	PWID (N = 57)
Identified pathogen, n (%)	40 (70.2%)
*Staphylococcus* spp., n (%)	38 (66.7%)
MRSA	14 (36.8%)
MSSA	24 (63.1%)
*Streptococcus* spp. (*pyogenes/agalactiae/dysgalactiae/gallolyticus/anginosus/constellatus/gordonii/mitis/oralis/salivarus/ sanguinis/viridans*), n (%)	8 (14%)
*Coxiella burnetii*, n (%)	0 (0%)
*Klebsiella spp*., n (%)	0 (0%)
*Enterococcus spp*., n (%)	1 (1.8%)
Acute fungal infection, n (%)	6 (10.5%)
Polymicrobial acute infection, n (%)	2 (3.5%)

Complications and outcomes

Identified complications were frequent and severe. Embolic events occurred in 58% of patients, mostly pulmonary but also cerebral and hepatic/splenic. Overall, 14% developed heart failure. Almost 30% required intensive care admission, and 25% had an indication for cardiac surgery, as described in Table 6.

**Table 6:** Clinical outcomes, hospitalization parameters, and mortality among PWID with IE (N = 57). SD = standard deviation; IQR = interquartile range; IE = infective endocarditis; PWID = people who inject drugs

Parameter	PWID (N = 57)
Intensive care unit admission, n (%)	17 (29.8%)
Cardiovascular surgery schedules, n (%)	14 (24.56%)
In-hospital mortality, n (%)	10 (17.5%)
Against medical advice discharge, n (%)	19 (33.3%)
Improved clinical status at discharge, n (%)	39 (68.4%)
Patient transfer to emergency hospitals, n (%)	3 (5.26%)
Hospitalization days	
Mean ± SD	29.49 ± 22.47
Median (IQR)	28 (9–44)
10-week mortality, n (%)	13 (22.8%)
12-month mortality, n (%)	20 (35.08%)

In-hospital mortality was 17.5%, increasing to 23% at 10 weeks and 35% at one year. The median hospital stay was 28 days. A third of the patients left the hospital against medical advice, while two-thirds were discharged with clinical improvement. Recurrence of endocarditis at least six months after discharge, during follow-up, was rare at less than 5% (4.87%).

Laboratory findings

On admission, patients showed high inflammatory activity, with elevated C-reactive protein (CRP), procalcitonin, and fibrinogen. These markers significantly decreased during hospitalization, while platelet counts improved. Hemoglobin levels remained low, and renal and liver function tests showed only a little change (Table 7).

**Table 7:** Laboratory data at admission and discharge among PWID with IE (N = 57). IE = infective endocarditis; PWID = people who inject drugs; CRP = C-reactive protein; ESR = erythrocyte sedimentation rate; CK-MB = MB isoenzyme of creatine kinase; GPT/ALT = glutamate-pyruvate transaminase/alanine aminotransferase; GOT/AST = glutamic oxaloacetic transaminase/aspartate aminotransferase; LDH = lactate dehydrogenase

Parameter	PWID (N = 57)
Leukocytes (×10³/µL) at admission, median (IQR)	11.9 (7.9–16.51)
Leukocytes (×10³/µL) at discharge, median (IQR)	9.8 (5.8–15.65)
CRP at admission (mg/dL), median (IQR)	12.7 (7.94–18.45)
CRP at discharge (mg/dL), median (IQR)	2.86 (0.68–10.45)
Procalcitonin at admission (mg/dL), median (IQR)	2.65 (0.42–10)
Procalcitonin at discharge (mg/dL), median (IQR)	0.11 (0.05–1.17)
Fibrinogen at admission (mg/dL), median (IQR)	430 (325.5–571.5)
Fibrinogen at discharge (mg/dL), median (IQR)	371.5 (298.75–461)
ESR at 1 hour from admission (mm/hour), median (IQR)	51 (38.5–70.5)
ESR at 1 hour at discharge (mm/hour), median (IQR)	50 (34.25–63)
Hemoglobin at admission (g/dL), median (IQR)	9.8 (8.95–10.85)
Hemoglobin at discharge (g/dL), median (IQR)	9.9 (5.9–14.3)
Trombocytes at admission (×10³/µL), median (IQR)	106 (56–247.15)
Trombocytes at discharge (×10³/µL), median (IQR)	212 (62.4–356.3)
Prothrombin index (%) at admission, median (IQR)	72 (64–80.5)
Prothrombin index (%) at discharge, median (IQR)	84 (66.25–92.5)
D-dimer at admission (mg/L), median (IQR)	3.5 (1.3–5.5)
D-dimer at discharge (mg/L), median (IQR)	2.4 (1.2–4.35)
Ultrasensitive troponin I at admission (TnI) (ng/mL), median (IQR)	55.7 (22.8–145.7)
Ultrasensitive Troponin I at discharge (TnI) (ng/mL), median (IQR)	32.5 (17.5–88)
CK-MB at admission (UI/L), median (IQR)	28.1 (16.75–53.87)
CK-MB at discharge (UI/L), median (IQR)	27 (16.5–43)
K^+^ at admission (mEq/L), median (IQR)	3.7 (3.3–4.1)
K^+^ at discharge (mEq/L), median (IQR)	4.1 (3.8–4.6)
Na^+^ at admission (mEq/L), median (IQR)	134 (129–137.5)
Na^+^ at admission (mEq/L), median (IQR)	137.6 (134–139)
Glycemia at admission (mg/dL), median (IQR)	101 (89–117.5)
Glycemia at admission (mg/dL), median (IQR)	88 (76–98.75)
Creatinine at admission (mg/dL), median (IQR)	0.9 (0.7–1.13)
Creatinine at discharge (mg/dL), median (IQR)	0.81 (0.7–1.09)
GPT/ALT at admission (UI/L), median (IQR)	29 (21–57.5)
GPT/ALT at discharge (UI/L), median (IQR)	37 (21–65)
GOT/AST at admission (UI/L), median (IQR)	56 (27–77.5)
GOT/AST at discharge (UI/L), median (IQR)	50.5 (33.62–88.75)
LDH at admission (UI/L), median (IQR)	310 (229.5–467.5)
LDH at discharge (UI/L), median (IQR)	298 (211–404)
Total bilirubin at admission (mg/dL), median (IQR)	0.9 (0.55–1.15)
Total bilirubin at discharge (mg/dL), median (IQR)	0.9 (0.8–1.1)

**Figure 5:**
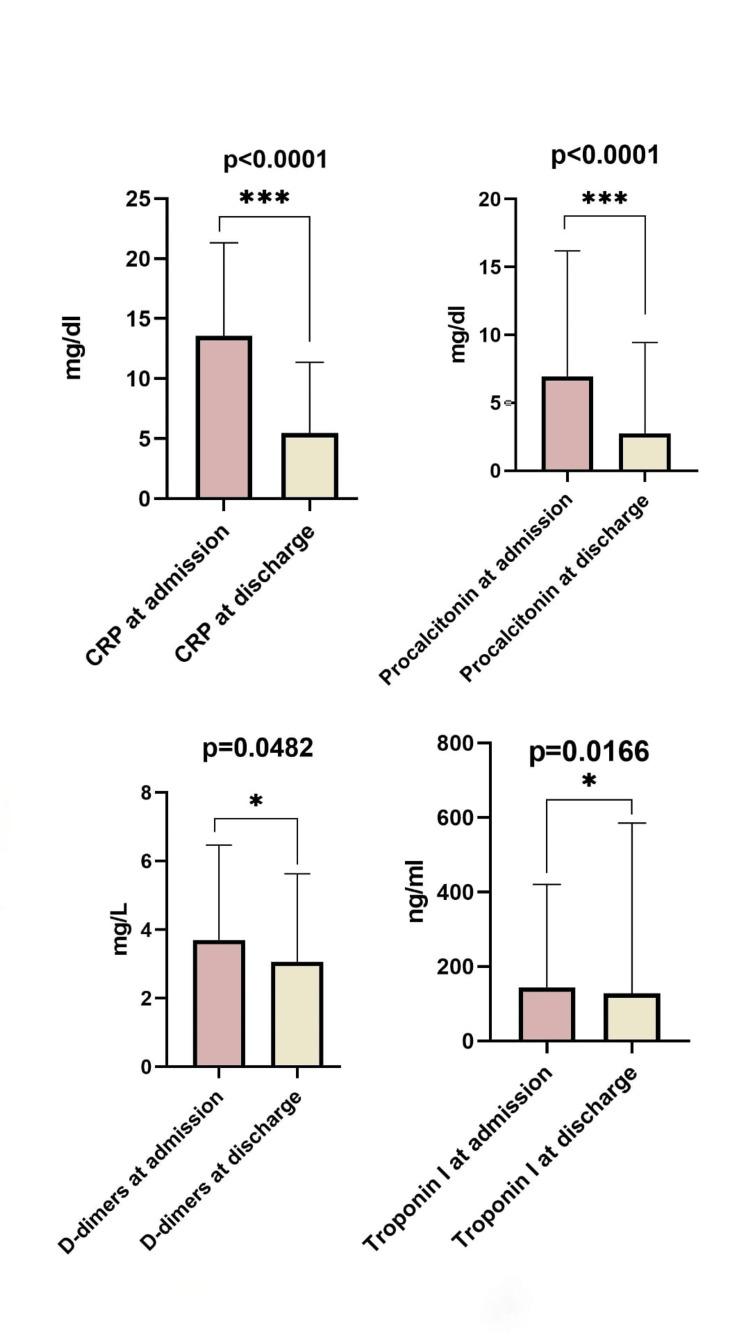
Laboratory findings: significant pair-wise comparisons at admission versus discharge. *: p < 0.05; **: p < 0.01; ***: p < 0.001. The Wilcoxon matched-pairs signed rank test was performed. A value of *p < 0.05 *is considered statistically significant. CRP = C-reactive protein

The Wilcoxon matched-pairs signed rank test was performed to observe which laboratory findings were significantly different at discharge from patient admission (Table 8, Figure 5).

**Table 8:** Paraclinical laboratory data changes during hospitalization. Wilcoxon matched-pairs signed ranked test. A p-value <0.05 is considered statistically significant. CRP = C-reactive protein; ESR = erythrocyte sedimentation rate; CK-MB = MB isoenzyme of creatine kinase; GPT/ALT = glutamate-pyruvate transaminase/alanine aminotransferase; GOT/AST = glutamic oxaloacetic transaminase/aspartate aminotransferase; LDH = lactate dehydrogenase

Laboratory changes during hospitalization	*P-value* (Wilcoxon matched-pairs signed ranked test)
Leukocytes	0.357
CRP	<0.001
Procalcitonin	<0.001
Fibrinogen	0.003
ESR	0.391
Hemoglobin	0.538
Thrombocytes	<0.001
PI (%)	<0.001
D-dimer	0.049
Ultrasensitive troponin I	0.017
CK-MB	0.870
K^+^	0.002
Na^+^	<0.001
Glycemia	0.001
Creatinine	0.447
GPT/ALT	0.381
GOT/AST	0.316
LDH	0.178
Total bilirubin	0.102

## Discussion

In our cohort of 57 PWID with IE, we saw a young group, largely male, with a median age of 34 years. This matches what many studies describe: PWID tend to be younger and have fewer baseline health issues than non-PWID with IE, but face more severe outcomes (e.g., right-sided infections, higher recurrence) [1,5,7,8,12-16]. We also noted extremely high rates of HIV (63%) and HCV (98%), much higher than in many Western cohorts, where HCV is common but HIV is less so [13,17,18]. These co-infections likely make managing IE even more complex and may contribute to worse outcomes.

Clinically, most patients had fever, fatigue, and respiratory symptoms, and echocardiography confirmed vegetations in over 90% of the patients. The tricuspid valve was most often involved (68%), which aligns with the classic right-sided pattern seen in PWID IE [13-15]. We had positive blood cultures in 70%, with *Staphylococcus aureus* dominating again, consistent with the literature [8,15].

Complications were frequent and serious, with more than half experiencing embolic events, mostly pulmonary. Some required intensive care unit admission (30%) or surgical intervention (25%). Long-term mortality was high at 35% at one year, which resonates with findings from multiple studies showing poor long-term outcomes despite the younger age of PWID [5,7,16].

For instance, in a meta-analysis of surgical outcomes of PWID with IE, one-year survival was about 81%, declining to 62% at five years and 57% at 10 years. PWID had both higher mortality and higher reoperation rates than non-PWID [8]. Another prospective cohort showed more embolic events and higher long-term mortality in PWID compared to matched non-users, even after adjusting for risk factors [19].

During hospitalization, there were several significant changes in laboratory values. Markers of systemic inflammation (CRP, procalcitonin, and fibrinogen) decreased clearly, which is consistent with a response to antimicrobial therapy. In contrast, leukocyte count and erythrocyte sedimentation rate did not change much, which suggests a slower normalization of these markers. Platelet count and platelet index increased, which fits with clinical improvement, while hemoglobin remained largely unchanged, which is common in patients with chronic disease or malnutrition. We also noted modest decreases in D-dimer and ultrasensitive troponin I, suggesting some recovery of coagulation balance and myocardial stress. Electrolytes (sodium and potassium) normalized during treatment. Renal and liver parameters, including creatinine, liver enzymes (alanine transaminase, aspartate transaminase), lactate dehydrogenase, and bilirubin, showed no major variation and were overall stable. Blood glucose also decreased, probably reflecting a reduction in stress hyperglycemia once infection was controlled.

Our study adds to these findings by highlighting the severity and recurring characteristics of IE, especially in PWID (especially because of the persistence of IV drug use), who are already at a high risk for infectious comorbidities. We also concluded that approximately one-third of our patients were discharged because of personal reasons, against medical advice, stressing the need for integrated care that includes addiction management and psychosocial support to prevent re-hospitalizations and overall mortality [14].

Limitations

This study has some limitations. First, because it is based on one tertiary infectious disease center, we probably see more severe cases than a general population sample, so selection bias is likely, and the findings may not generalize well to the community. Second, follow-up was variable, and for some patients, we simply could not obtain long-term information, which means there is a risk of attrition bias. Third, missing data were handled as available-case analysis, and Charlson and SOFA scores were calculated from the parameters documented at admission. Moreover, the long inclusion period implies that clinical practice, substance use patterns, and even diagnostic routines may have changed over time. Finally, as this was a descriptive study, we did not adjust for confounders, and the associations presented should be interpreted cautiously, more as observations than causal estimates. Even so, we believe the study adds useful information on IE in PWID in an Eastern European context, a group that is not well represented in published data.

## Conclusions

Overall, PWID with IE in our cohort showed severe disease, frequent complications, and high mortality, despite prolonged hospital care. These findings emphasize the fact that treating IE in this particular population at risk cannot rely solely on antibiotic therapy and surgery. Progress will be made by adjusting this therapeutic approach into an integrated one that brings together infection management, addiction treatment, and mental health support that addresses the person as a whole. By combining medical expertise with social, economic, and psychological care, we hope to achieve long-term survival and quality of life for these vulnerable patients.
